# The influence of chronic renal failure on the spectrum and antimicrobial susceptibility of uropathogens in community-acquired acute pyelonephritis presenting as a positive urine culture

**DOI:** 10.1186/1471-2334-11-102

**Published:** 2011-04-21

**Authors:** Yeon Soon Jung, Ho Sik Shin, Hark Rim

**Affiliations:** 1 Department of Internal Medicine, Kosin University College of Medicine, Busan, Republic of Korea

**Keywords:** Pyelonephritis, Chronic Renal Failure, Antimicrobial Susceptibility

## Abstract

**Background:**

The role of chronic renal failure (CRF) in the antimicrobial resistance of uropathogens in patients with community-acquired acute pyelonephritis (APN) remains poorly understood.

**Method:**

We performed a retrospective analysis of 502 adults (54 men, 448 women; mean age 61.7 ± 16.0 years, range 18-98 years) who were treated for community-acquired APN at Kosin University Gospel Hospital (Busan, Republic of Korea) during a ten-year period (January 2000 to December 2009). We evaluated the spectra and antimicrobial susceptibility profiles of uropathogens in CRF and non-CRF patients with community-acquired APN that presented as a positive urine culture.

**Results:**

The 502 adult subjects were classified as either non-CRF APN patients (336 patients, 66.9%) or CRF APN patients (166 patients, 33.1%) according to their estimated glomerular filtration rate. No significant differences in the sensitivity of *E. coli *to a third cephalosporin, aminoglycoside (except gentamycin), or ciprofloxacin were observed between non-CRF and CRF patients.

**Conclusions:**

In our series of patients with community-acquired APN that initially presented as a positive urine culture, CRF did not influence the isolation rates of different uropathogens or their patterns of susceptibility to antimicrobials.

## Background

Acute pyelonephritis (APN), an infectious disease of the renal parenchyma and pelvic region, is a significant and frequent cause of morbidity, resulting in more than 100,000 hospital admissions per year in the United States [1,2]. The most common pathogens in APN belong to the Enterobacteriaceae family, and *Escherichia coli *is the causative pathogen in more than 80% of cases [1]. Other microbes contributing to the pathogenesis of APN include *Proteus *spp, *Klebsiella *spp, and enterococci [3]. Risk factors that predispose women to APN include diabetes mellitus (DM), incontinence, patient and family history of urinary tract infections (UTIs), and certain sexual behaviors [4]. Although DM is a risk factor, it was previously reported that DM does not influence the isolation rates of different uropathogens or their patterns of susceptibility to antimicrobials [5].

In general, CRF patients are known to be vulnerable to infection due to weakened immunity [6]. But, few data are available on the role of CRF as a risk factor for the development of antimicrobial resistance of the uropathogens [7]. For this reason, we have undertaken a study to evaluate the spectrum of etiologic agents and their profiles of antimicrobial resistance in a large series of non-CRF and CRF patients with community-acquired APN that presented as a positive urine culture.

## Methods

### Patients

We performed a retrospective analysis of the medical records of Korean adults who were admitted to Kosin University Gospel Hospital (Busan, Republic of Korea) due to community-acquired APN between January 2000 and December 2009 and evaluated the spectra and antimicrobial susceptibility profiles of uropathogens in both non-CRF and CRF patients with community-acquired APN that presented as a positive urine culture. The analysis included 502 adults aged 18 years or older (54 men, 448 women; mean age 61.7 ± 16.0 years, range 18-98 years).

### Inclusion and Exclusion Criteria

Our clinical diagnosis of APN was based on the study of Safrin et al. (1988) [8]. In addition to the clinical diagnosis, which was based on signs and symptoms of APN in adult patients, patients were required to meet at least one of the following three criteria: 1) a positive dipstick test for leukocyte esterase; 2) a white blood cell (WBC) count ≥ 5 cells per high-power field microscopy on examination of centrifuged urine sediment; or 3) a WBC count ≥ 10 cells/ μL in non-centrifuged urine.

The exclusion criteria were as follows: 1) less than 18 years of age; 2) history of chronic infection; 3) evidence of other infection; 4) previous administration (in the previous six months) of antibiotics; 5) urologic abnormalities; 6) hospital-acquired APN (infection acquired during hospital care which was not present or incubating at time of admission. Infections occurring more than 48 hours after admission are usually considered nosocomial) [9]; 7) presence of an indwelling bladder catheter; 8) anticancer therapy or immunosuppressive medication, such as steroids; 9) pregnancy; and 10) negative results on urine culture.

### Clinical and biochemical assessment

Type 2 diabetes was diagnosed according to the Report of the Expert Committee on the Diagnosis and Classification of Diabetes Mellitus [10].

As a surrogate for renal function, we estimated the glomerular filtration rate (GFR) using a simplified form of the Modification of Renal Disease (MDRD) equation [11]:

CRF was defined as follows [12]: eGFR less than 60 mL/min/1.73 m^2 ^without kidney damage for more than three months.

Baseline urine specimens were collected using a sterile, midstream, clean-catch technique. Specimens were sent to a central laboratory for urinalysis and susceptibility testing, and a urine culture was performed on each sample. Central laboratories were required to use the methodology of the Clinical and Laboratory Standards Institute (CLSI) [13] with quality-control methods using appropriate American Type Culture Collection organisms. Quantitative urine culture was performed using a dipslide method; urine was also streaked onto MacConkey agar. After incubation at 37°C for 24 h, the microorganisms were identified using standard biochemical tests. Presence of a pathogen in the urine culture was confirmed based on a colony count of greater than or equal to 10^5 ^CFU/mL. *In vitro *susceptibility to antibiotics was performed using an agar diffusion method (Kirby Bauer) employing dried filter paper discs impregnated with specific concentrations of antimicrobial agents, in accordance with the National Committee for the Clinical Laboratory Standards [14]. Susceptibility testing results for pathogens present upon admission were categorized as *susceptible *or *resistant*, and susceptibility guidelines were taken from the CLSI guidelines [13]. We also investigated antibiotic sensitivities to ampicillin, cephalothin, cefuroxime, cefotaxime, cefazolin, ceftazidime, imipenem, gentamycin, amikacin, tobramycin, ciprofloxacin, and trimethoprim-sulfamethoxazole (TMP-SMX)

### Statistical analysis

The results are presented as the mean ± SD. We used Student's *t *test to compare the means between groups. Differences in antibiotic sensitivity and uropathogen profiles between groups were analyzed using the *X*^2 ^test. The results were considered significant when the *P *value was less than 0.05. All statistical analyses were performed using the Statistical Package for the Social Sciences (SPSS), version 12.0 (SPSS Inc, Chicago, IL, USA).

## Results

The characteristics of the 502 subjects enrolled in this study are shown in Table 1. The mean age was 61.7 (± 16.0) years, with 89.2% of the subjects being women and 20.1% being diabetic patients.

**Table 1:** Age-gender standardized baseline demographics and laboratory results according to eGFR

eGFR	≥ 60 mL/min/1.73 m^2^, n = 336	< 60 mL/min/1.73 m^2^, n = 166	p-value
Characteristics			
**Age, years**	59.0 ± 16.8	67.3 ± 12.4	0.0001
**Genders, %**			0.168
Males	12.2	7.8	
Females	87.8	92.2	
**Diabetes, %**	20.8	21.4	0.906
**Measurements**			
WBC, mm^3^	9,944 ± 5,630	11,882 ± 6,233	0.001
Hb, g/dL	11.5 ± 1.9	11.1 ± 1.9	0.083
Platelet, mm^3^	226,232 ± 106,223	212,380 ± 112,614	0.182
BUN, IU/L	12.4 ± 6.0	27.4 ± 14.4	0.0001
Creatinine, IU/L	0.72 ± 0.15	1.62 ± 0.64	0.0001
eGFR, mL/min/1.73 m^2^	98 ± 32	39 ± 13	0.0001
Sodium, mEq/L	136 ± 5	135 ± 6	0.157
Potassium, mEq/L	3.8 ± 0.5	4.0 ± 0.7	0.003
Protein, g/dL	6.6 ± 3.6	6.3 ± 0.9	0.334
Albumin, g/dL	3.4 ± 0.6	3.2 ± 0.6	0.002
Total Bilirubin, mg/dL	1.36 ± 2.10	1.65 ± 2.14	0.172
Direct Bilirubin, mg/dL	0.74 ± 1.61	0.93 ± 1.50	0.233
AST, IU/L	47.2 ± 76.9	72.4 ± 249.3	0.216
ALT, IU/L	39.0 ± 71.6	37.2 ± 87.2	0.813
ALP, IU/L	130.5 ± 130.3	124.7 ± 133.6	0.675
r-GTP, IU/L	110.3 ± 202.9	82.9 ± 115.2	0.085
PT, second	92.5 ± 21.4	84.2 ± 22.5	0.001
PT INR	1.09 ± 0.29	1.19 ± 0.65	0.048
PTT, second	37.8 ± 13.5	39.8 ± 10.9	0.133
CRP, mg/dL	6.6 ± 7.4	8.8 ± 8.5	0.045
HS-CRP, mg/dL	7.0 ± 7.8	11.0 ± 9.4	0.002
Total cholesterol, mg/dL	163 ± 47	154 ± 49	0.180
HDL cholesterol, mg/dL	41 ± 16	36 ± 17	0.032
LDL Cholesterol, mg/dL	102 ± 40	85 ± 38	0.008
Triglycerides, mg/dL	105 ± 60	131 ± 107	0.041
Chloride, mEq/L	100 ± 5	101 ± 7	0.575
total CO_2_, mEq/L	26.7 ± 4.8	22.7 ± 5.6	0.0001
ESR, mm/hour	30.5 ± 23.7	36.2 ± 23.0	0.030
Urine RBC (0-4+)	1.32 ± 2.22	1.36 ± 1.91	0.003
Urine protein (0-4+)	0.65 ± 0.89	0.83 ± 1.00	0.0001
Urine WBC (0-4+)	2.29 ± 2.62	2.99 ± 2.76	0.0001

The etiologic microorganisms of APN are shown in Table 2. The most common cause of APN was *E. coli *(58.3%, 293 cases), followed by *K. pneumonia *(12.7%, 64 cases), *Pseudomonas *(4.1%, 21 cases), and *Enterococcus *(2.9%, 15 cases).

**Table 2:** Etiologic Microorganisms of APN

Pathogen	Male	Female	Total
***E.coli***	12	281	293
***K.pneumoniae***	8	56	64
***Proteus***	3	11	14
***Pseudomonas***	9	12	21
***Enterococcus***	2	13	15
**G(+)**	16	50	66
**G(-)**	4	25	29
**Total**	54	448	502

APN: Acute Pyelonephritis, G(+): Gram positive microorganism

G(-): Gram negative microorganism

The antimicrobial sensitivity rates for *E. coli *are shown in Table 3. The sensitivity rates were 100% for imipenem, 99.2% for amikacin, 90.8% for tobramycin, over 80% for third- and fourth-generation cephalosporin antibiotics, 71.7% for ciprofloxacin, and 61.4% for TMP-SMX. However, the sensitivity rates for gentamycin and ampicillin were low (42.9% and 39.4%, respectively).

**Table 3:** Antimicrobial Sensitivity Rate (%) for *E.coli *according to eGFR

Antibiotics	≥ 60 mL/min/ 1.73 m^2^	< 60 mL/min/ 1.73 m^2^	p-value
**Ampicillin**	37.8	20.0	0.024
**Cephalothin**	45.5	36.0	0.428
**Cefuroxime**	76.9	69.4	0.320
**Cefotaxime**	82.0	76.5	0.413
**Cefozolin**	66.7	47.1	0.162
**Ceftazidime**	83.2	75.5	0.259
**Cepefime**	83.6	80.4	0.613
**Imipenem**	95.5	100	0.126
**Gentamycin**	71.9	51.9	0.016
**Amikacin**	96.3	90.0	0.112
**Tobramycin**	87.0	82.0	0.404
**Ciprofloxacin**	72.7	66.0	0.389
**Trimethoprime-sulfomethoxazole**	57.9	56.9	0.905

Age-gender standardized baseline demographics and laboratory results according to eGFR are shown in Table 1. The rates of diabetes in the non-CRF and CRF groups were 20.8% and 21.4%, respectively (p = NS). The mean ages were 59.0 (± 16.8) years in the non-CRF group and 67.3 (± 12.4) years in the CRF group (p = 0.0001), and the values of HS-CRP were 7.0 (± 7.8) mg/dL in the non-CRF group and 11.0 (± 9.4) mg/dL in the CRF group (p = 0.002). The values for WBC (p = 0.001) and ESR (p = 0.03) were higher in the CRF group than in the non-CRF group.

The antimicrobial sensitivity rates (%) for *E. coli *according to eGFR are shown in Table 3. The antimicrobial sensitivities to ampicillin in the non-CRF group and CRF group were 37.8% and 20.0%, respectively (p = 0.024), and sensitivities to gentamycin in the non-CRF group and CRF group were 71.9% and 51.9% (p = 0.016), respectively. Significantly higher resistance of *E. coli *to amoxicillin and gentamycin was noted in isolates recovered in patients with CRF. No significant differences in the sensitivity of *E. coli *were observed between non-CRF and CRF patients.

The isolation rates (%) of uropathogens in APN patients with or without CRF are shown in Table 4. The isolation rates (%) for *E. coli *in the non-CRF group and CRF group were 54.8% and 65.7% (p = NS), and the isolation rates for *K. pneumoniae *were 12.2% and 13.9%, respectively (p = NS). The isolation rates of other microorganisms did not differ between the two groups.

**Table 4:** Isolation rate (%) of uropathogens in APN patients with or without CKD

Uropathogens	Non-CKD	CKD	p-value
***E. coli***	54.8	65.7	NS
***K. pneumoniae***	12.2	13.9	NS
***Proteus***	2.7	3.0	NS
***Pseudomonas spp***	5.1	2.4	NS
***Enterococcus spp***	3.0	3.0	NS
**Other gram positive**	15.2	9.0	NS
**Other gram negative**	7.1	3.1	NS

The antimicrobial sensitivity rates (%) for *E. coli *according to DM are shown in Table 5. The antimicrobial sensitivities to ampicillin in the non-DM group and DM group were 30.6% and 35.7%, respectively (p = 0.538), and sensitivities to gentamycin in the non-DM group and DM group were 67.2% and 63.4% (p = 0.656), respectively. The antimicrobial sensitivities to other antibiotics did not differ between the two groups.

**Table 5:** Comparison between diabetes mellitus and non-diabetes mellitus patients with respect to antimicrobial sensitivity rate (%) for *E. coli*

Antibiotics	Non-DM	DM	p-value
**Ampicilline**	30.6	35.7	0.538
**Cephalothin**	36.7	52.9	0.227
**Cefuroxime**	72.2	78.4	0.456
**Cefotaxime**	78.2	84.2	0.420
**Cefozolin**	55.3	81.8	0.106
**Ceftazidime**	80.0	80.6	0.942
**Cepefime**	81.4	84.2	0.690
**Imipenem**	95.7	100	0.195
**Gentamycin**	67.2	63.4	0.656
**Amikacin**	92.2	100	0.081
**Tobramycin**	84.5	86.5	0.767
**Ciprofloxacin**	71.2	62.6	0.674
**Trimethoprime-sulfomethoxazole**	56.8	61.0	0.638

## Discussion and Conclusions

In this study, we attempted to determine whether there are differences between CRF and non-CRF patients in the bacteriologic patterns of community-acquired APN or in the antibiotic sensitivity patterns of uropathogens. This study showed that CRF does not seem to influence the isolation rates of different uropathogens or their susceptibility patterns to antimicrobials in patients with community-acquired APN that presented as a positive urine culture.

In general, CRF patients are known to be vulnerable to infection due to weakened immunity [6]. But little information is available regarding the role of CRF as a risk factor for the development of antimicrobial resistance in uropathogens. It has been reported that DM *per se *does not seem to influence the isolation rates of different uropathogens or their susceptibility patterns to antimicrobials [5]. In this study, the antimicrobial susceptibilities of microorganisms isolated in cases of community-acquired APN in non-DM and DM groups did not differ. However, the role of CRF in the etiology and antimicrobial resistance of uropathogens in patients with community-acquired APN has not been clarified.

It is known that *E. coli *is isolated in approximately 90% of APN cases [15]. Other studies have found that urinary Klebsiella is more frequent in diabetic patients compared to the detection rate in non-diabetic patients [16,17]. In this study, the most common cause of APN was *E. coli *(58.3%, 293 cases), followed by *K. pneumoniae *(12.7%, 64 cases). Due to the inclusion of diabetic patients, *K. pneumoniae *was more common than in previous reports on APN. Among the microorganisms associated with APN, *E. coli *was found in 54.8% and 65.7% of cases in the non-CRF group and CRF group, respectively (p = NS), and *K. pneumonia *was present in 12.2% and 13.9% of cases, respectively (p = NS). There were no differences between the groups regarding the rates of other APN microorganisms.

In a previous analysis of the antibiotic sensitivity of *E. coli *in APN, the sensitivities to the first- and third-generation cephalosporins, aminoglycoside and ciprofloxacin were greater than 90%; 47% to ampicillin; and 60% to TMP-SMX [18]. In our current study, the rate of *E. coli *sensitivity was 96.9% to imipenem, 99.2% to amikacin, 90.8% to tobramycin, greater than 80% to third- and fourth-generation cephalosporin antibiotics, 71.7% to ciprofloxacin, and 61.4% to TMP-SMX. However, the sensitivity rates to gentamycin and ampicillin were low (42.9% and 39.4%, respectively). Little data is available on the role of CRF as a risk factor for the development of antimicrobial resistance in uropathogens. Antimicrobial sensitivities to ampicillin in the non-CRF group and CRF group were 37.8% and 20.0%, respectively (p = 0.024), and those to gentamycin in the non-CRF group and CRF group were 71.9% and 51.9%, respectively (p = 0.016). The antimicrobial sensitivities to other antibiotics did not differ between the two groups. In North America, a cut-off point of 20% has been suggested as the level of resistance at which an agent should no longer be used [19]. The observed high rates of *E. coli *resistance to ampicillin, cephalothin, and gentamycin precludes, at least in our area, the choice of these or similar drugs in the empirical initial treatment of community-acquired APN in CRF and non-CRF patients.

In a recent case-control study, recent hospitalization and fluoroquinolone use in the previous six months were independent risk factors for fluoroquinolone resistance in community-onset febrile *E. coli *UTI [20]. In our study, we excluded cases of previous administration of antibiotics and hospital-acquired APN.

Recent cohort studies have suggested that CRF is also a risk factor for non-cardiovascular morbidity [21] and mortality, including those caused by infection [22,23]. Few studies have investigated the associations between CRF and specific infectious conditions. In this study, the value of HS-CRP was 7.0 (± 7.8) mg/dL in the non-CRF group and 11.0 (± 9.4) mg/dL in the CRF group (p = 0.002). The values of WBC (p = 0.001) and ESR (p = 0.03) were higher in the CRF group than they were in the non-CRF group. Based on these results, we propose that patients with CRF had higher values of inflammatory markers when they had APN and that more attention is needed in this area because community-acquired APN in CRF patients can be a serious illness.

The limitations of our study include performance on non-CRF and CRF patients admitted to a single hospital, although the study included a large number of patients. Second, the CRF group was older in age than was the non-CRF group. Finally, the results for blood culture were not included in this study.

Based on our results, ampicillin, cephalothin, and gentamycin should not be considered as an initial therapeutic regimen in either CRF or non-CRF patients with community-acquired APN. In our series of patients with community-acquired APN presenting with a positive urine culture test, CRF *per se *does not seem to influence the isolation rates of different uropathogens or their susceptibility patterns to antimicrobials. A detailed prospective study is required to address the influences of CRF on the spectrum and antimicrobial susceptibility of the uropathogens involved in community-acquired APN.

## Competing interests

This study was supported by a grant from the Kosin University College of Medicine (2010).

## Authors' contributions

YSJ, M.D. and HSS, M.D. participated in the design of the study and performed the statistical analysis. HR, M.D. conceived of the study, and participated in its design and coordination. All authors read and approved the final manuscript.

## Pre-publication history

The pre-publication history for this paper can be accessed here:

http://www.biomedcentral.com/1471-2334/11/102/prepub
